# Does abstaining from alcohol in high school moderate intervention effects for college students? Implications for tiered intervention strategies

**DOI:** 10.3389/fpsyg.2022.993517

**Published:** 2022-11-30

**Authors:** Lin Tan, Zachary Friedman, Zhengyang Zhou, David Huh, Helene R. White, Eun-Young Mun

**Affiliations:** ^1^School of Public Health, The University of North Texas Health Science Center at Fort Worth, Fort Worth, TX, United States; ^2^Center of Alcohol and Substance Studies, Rutgers University, New Brunswick, NJ, United States; ^3^School of Social Work, University of Washington, Seattle, WA, United States

**Keywords:** brief alcohol intervention, adolescent drinking, personalized normative feedback, alcohol misuse, moderator analysis, college students

## Abstract

Brief motivational intervention (BMI) and personalized feedback intervention (PFI) are individual-focused brief alcohol intervention approaches that have been proven efficacious for reducing alcohol use among college students and young adults. Although the efficacy of these two intervention approaches has been well established, little is known about the factors that may modify their effects on alcohol outcomes. In particular, high school drinking may be a risk factor for continued and heightened use of alcohol in college, and thus may influence the outcomes of BMI and PFI. The purpose of this study was to investigate whether high school drinking was associated with different intervention outcomes among students who received PFI compared to those who received BMI. We conducted moderation analyses examining 348 mandated students (60.1% male; 73.3% White; and 61.5% first-year student) who were randomly assigned to either a BMI or a PFI and whose alcohol consumption was assessed at 4-month and 15-month follow-ups. Results from marginalized zero-inflated Poisson models showed that high school drinking moderated the effects of PFI and BMI at the 4-month follow-up but not at the 15-month follow-up. Specifically, students who reported no drinking in their senior year of high school consumed a 49% higher mean number of drinks after receiving BMI than PFI at the 4-month follow-up. The results suggest that alcohol consumption in high school may be informative when screening and allocating students to appropriate alcohol interventions to meet their different needs.

## Introduction

In a 2020 national survey, 56% of full-time college students who were 1 to 4 years beyond high school reported past 30-day drinking, and 24% reported five or more drinks in a row in the past 2 weeks (Schulenberg et al., 2021). The high prevalence of alcohol misuse in college populations is associated with a greater likelihood of alcohol-related adverse events, including assault, unsafe sex, academic problems, and death (Hingson et al., 2017). College environments provide increased opportunities for alcohol consumption (Schulenberg et al., 2004); however, many young adults initiate drinking before college (Richmond-Rakerd et al., 2017). In 2021, 26% of 12^th^ graders reported past-month drinking, and 12% reported past-month binge drinking (Johnston et al., 2022). High school drinking matters because those with drinking experience prior to college tend to consume greater quantities of alcohol in college (Arria et al., 2008; Stappenbeck et al., 2010), whereas those who abstain from drinking during the last year of high school are more likely to stay abstinent in college (Huang et al., 2011).

First-year college students with a history of drinking may be more likely to associate drinking with positive consequences, including enhanced relaxation, reduced stress, and increased sociability (Lee et al., 2018a), and continue drinking in college to upregulate positive affect and down-regulate negative affect. After entering college, students may quickly self-organize into social environments that match their drinking behaviors and allow for more drinking opportunities (Sher and Rutledge, 2007; Park et al., 2009). Consequently, students who drank in high school tend to continue or increase drinking in college with little or no motivation to change their drinking behaviors (Qi et al., 2014). In contrast, students who start drinking after the transition to college may commonly do so in the spirit of exploration. College is a formative period to experience new behaviors, test preferences, and formulate values (Dworkin, 2005). For many, experimentation with risky behaviors is expected to a certain extent and may not necessarily lead to problematic drinking behaviors. More typically, college students may gradually mature out of heavy drinking patterns after a temporary spike in alcohol use upon entering college (Windle, 2020), especially if they are made aware of the actual norms of how their peers drink and/or the negative consequences of drinking (Cronce and Larimer, 2011). Compared to their peers with a history of drinking before college, students with no prior experience drinking may have fewer positive expectations regarding drinking and no established drinking habits; therefore, they might respond better to a brief alcohol intervention aimed at modifying their drinking behaviors. Unfortunately, there has been little empirical research examining whether and how high school drinking may be associated with different outcomes following brief alcohol interventions.

The primary goal of this work is to investigate the extent to which college students with and without a prior history of high school drinking may respond differently to brief alcohol interventions, more specifically, personalized feedback intervention (PFI) and brief motivational intervention (BMI). PFI provides personalized feedback designed to change alcohol use behaviors by increasing the salience of normative discrepancies, the awareness of personal alcohol use patterns, and the cognizance of negative consequences and risks associated with alcohol use (Cronce and Larimer, 2011). While also providing personalized feedback, BMI focuses on increasing students’ motivation for change by a counselor delivering the feedback in a supportive, nonconfrontational, and nonjudgmental style (Miller and Rollnick, 2002). Although available evidence on brief alcohol interventions suggests that both PFI and BMI are efficacious in reducing college students’ alcohol use and/or negative consequences (Cronce and Larimer, 2011; National Institute on Alcohol Abuse and Alcoholism, 2019), recent large-scale meta-analyses on comparative effectiveness found that BMI had a stronger effect on lowering alcohol-related problems (Huh et al., 2015; Jiao et al., 2020) and the risk of driving after four+/five+ drinks (Mun et al., 2022a) than PFI. In the main outcome study using the data from the current study, White et al. (2007) found no difference in the drinking outcomes between students randomized to a BMI vs. a PFI at the 4-month follow-up. At the 15-month follow-up, students randomized to the BMI reported lower levels of alcohol use and alcohol-related problems than those randomized to the PFI. However, it is unclear which intervention approach is better for college students with versus without a history of high school drinking.

The current study aims to extend the existing literature by examining the moderating effects of high school drinking (individual-level factor) on the effects of interventions (BMI vs. PFI) on alcohol use, which can help guide intervention decisions when personalizing and optimizing to better fit the needs of different students. Because BMI focuses on increasing readiness to change among college students who drink but may not be interested in changing their alcohol-related behaviors (Barnett et al., 2001), we hypothesized that BMI would have stronger intervention effects than PFI for students with a high school drinking history.

## Materials and methods

### Participants

**Figure 1:**
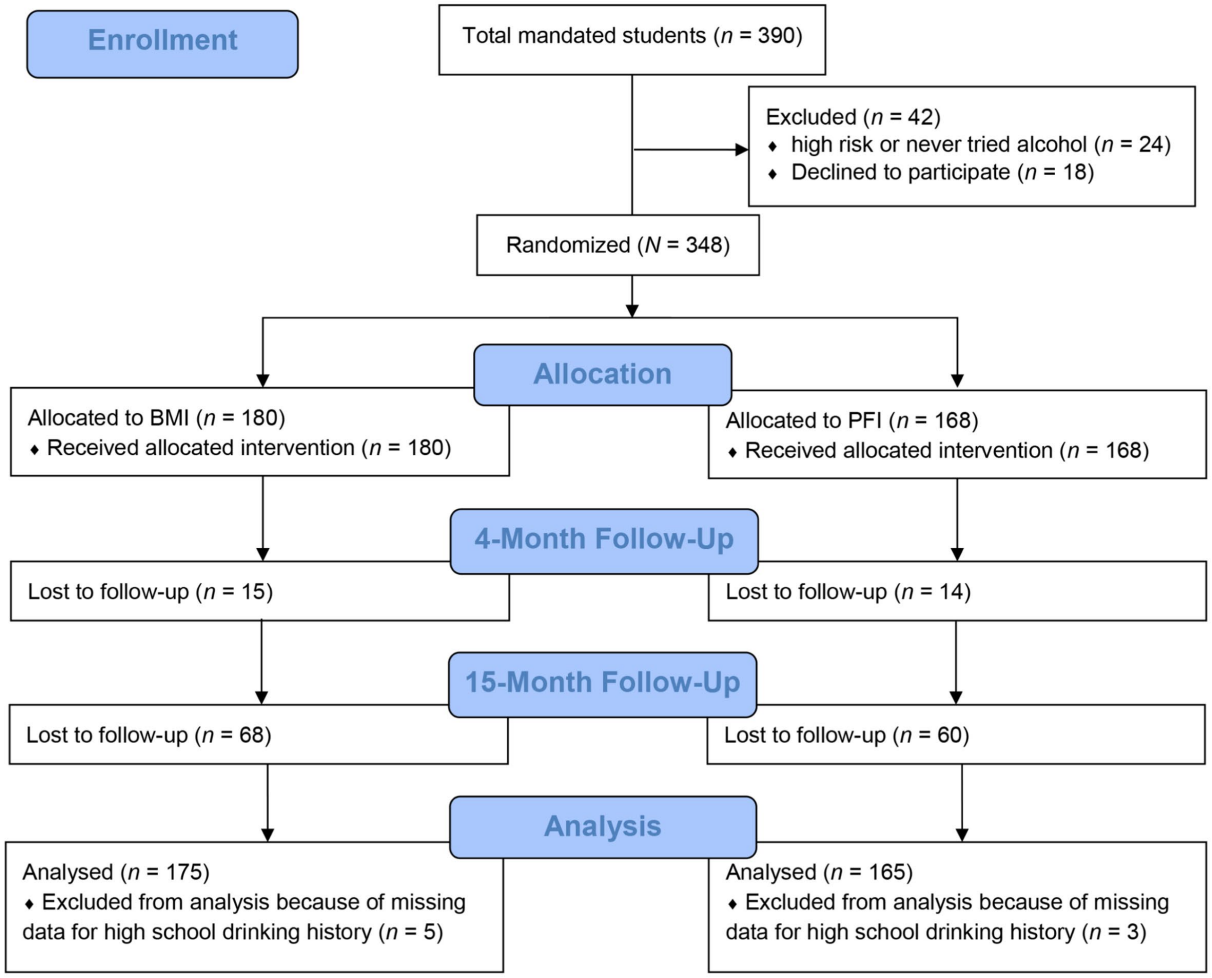
The CONSORT flow diagram.

Participants were students at a large public university who violated the university’s alcohol and drug use policy and thus were mandated to receive alcohol and drug use counseling at the university-based treatment center. A total of 390 students were mandated to treatment during the Fall 2003, Spring 2004, and Fall 2004 semesters. Of the mandated students, those who were deemed high-risk or those who had never tried alcohol were excluded from the larger study (for more detailed exclusion criteria, see White et al., 2007; see Figure 1 for a CONSORT flow diagram). The participants for the current study were 348 mandated students. The mean age at baseline was 18.6 (*SD* = 1.0). Most participants were first-year students (61.5%). See Table 1 for additional descriptive statistics of the sample.

**Table 1:** Descriptive statistics of the study variables.

	BMI (*n* = 180)		PFI (*n* = 168)	
	*M* (*SD*) or %	*% Zero*	*M* (*SD*) or %	*% Zero*
*Baseline*				
Male (coded 1; 0 = female)	57.8%		62.5%	
White (coded 1; 0 = non-White)	70.6%		76.2%	
First-year student (coded 1; 0 = non-first-year)	64.4%		58.3%	
High school drinking (1 = drank alcohol; 0 = did not drink at all)	80.6%		79.4%	
Number of drinks in a typical week	7.57 (6.87)	2.8%	7.05 (5.94)	8.3%
*4-month follow-up*				
Number of drinks in a typical week	5.08 (6.21)	18.9%	5.29 (7.18)	29.8%
*15-month follow-up*				
Number of drinks in a typical week	6.83 (7.80)	18.9%	8.27 (11.45)	18.5%

### Procedures

The study was approved by the Rutgers Institutional Review Board. All participants agreed to participate and signed informed consent forms. Students completed a baseline assessment questionnaire that focused on students’ drinking and alcohol use habits, which was used to generate personalized feedback. Approximately 1 week after the baseline assessment, eligible students were randomized either to a BMI (*n* = 180; 51.7%) or to a PFI condition (*n* = 168; 48.3%) by the flip of a coin. The individualized personal feedback was developed detailing the student’s alcohol and drug use behaviors, attitudes toward alcohol and drugs, and a variety of consequences associated with alcohol and drug use (e.g., academic difficulties and interpersonal problems) that they had experienced. The profile also focused on comparing the students’ alcohol and drug use to peer norms as well as providing information about blood alcohol concentrations, alcohol tolerance, and strategies for reducing risky behaviors (see Appendix A in the online supplemental material for a profile example).

Students in the PFI condition were handed the written feedback profile by a counselor and were asked to take it home with them to review but did not meet with the counselor to discuss the feedback. Those students in the BMI condition met individually with a counselor for a single session (approximately 30 min), during which they discussed their written feedback. The counselor provided feedback to the student based on the principles of motivational interviewing (Miller and Rollnick, 2002), with a focus on expressing empathy, developing discrepancy between the student’s alcohol and drug use and expressed values, respecting resistance, and supporting self-efficacy.

Follow-up surveys were conducted approximately 4 months following the intervention (*n* = 319, 91.7%), and again 15 months after the intervention (*n* = 220; 63.2%). At the 4-month follow-up, 76% of students completed a questionnaire in person; 20% completed a phone interview with the research staff; and 4% completed and mailed in questionnaires to the research office. At the 15-month follow-up, students either completed a secure website survey (57%) or a telephone interview (43%). No significant differences were found between those who were followed up and those who did not complete the follow-up interviews on demographic or baseline alcohol use variables. More details of the study procedures can be found in White et al. (2007), where the primary outcomes of the interventions are reported.

### Measures

#### Alcohol use outcome variables

Students reported the number of drinks they had in a typical week in the past month using the Daily Drinking Questionnaire (DDQ; Collins et al., 1985) at baseline, 4-and 15-month follow-ups.

#### Intervention group

An indicator variable identified the type of brief alcohol intervention that the participant received, with BMI coded as 1 and PFI coded as 0.

#### High school drinking history

Participants self-reported the frequency of alcohol use during their senior year of high school (1 = did not drink at all to 8 = once a day or more) at the baseline assessment, which was recoded into high school drinking in the current study (0 = did not drink at all [20% of students] and 1 = drank alcohol [80% of students]). The results using the original ordinal scale of high school drinking history are presented in Supplementary Table S1 in the online supplemental material.

#### Demographic variables

Sex (1 = male; 0 = female), race (1 = White; 0 = non-White), and year in college (1 = first-year; 0 = non-first-year) were included in the model as covariates as they are related to alcohol use (Mun et al., 2009).

### Data analysis plan

We merged the imputed data from White et al. (2007) with high school drinking history to create the data set used for the current study. For the analysis reported in White et al. (2007), a single imputed data set was obtained using the expectation–maximization algorithm in SAS version 9 (SAS Institute, 2002–2006). The imputed data set was larger in dimensions (i.e., variables) than the analyzed data set and had 14% missing data before imputation. Missing data revealed a nonsignificant Little’s (1988) chi-square test of Missing Completely at Random (MCAR), χ*^2^* (8,020) = 8,078.96, *ns*, suggesting that missing data would be ignorable for inference. Eight participants (2% of *N* = 348) with missing high school drinking history responses were excluded from the analyses in the current study. The outcome variable, the number of drinks participants consumed in a typical week, was a count variable with excessive zeros at both the 4-month and 15-month follow-ups. Because of the zero-inflation in the outcome variable, we utilized the Marginalized Zero-Inflated Poisson (MZIP; see Long et al., 2014 and Mun et al., 2022b, for detailed model specifications) model to evaluate our hypotheses. The MZIP model is a statistical approach for modeling zero-inflated count outcomes, based on the framework of the zero-inflated Poisson (ZIP) model, that can (a) account for excessive zeros and (b) estimate the effects of predictors on the overall mean for the entire distribution. The formulation of a ZIP model can be expressed as:


$$Y_{i}\sim \{\begin{array}{c} 0,\;\;\mathrm{with probability}\;\pi_{i}\;\\ \begin{array}{cc} Poisson\left(\mu_{i}\right), & \mathrm{with probability}\;1-\pi_{i} \end{array} \end{array}\;,$$

logπi1−πi=γ0ZIP+γ1ZIP·Intervention+γpZIP∗AdditionalCovariates,

and


logμi=β0ZIP+β1ZIP·Intervention+βpZIP∗AdditionalCovariates,

where $\pi_{i}=Prob\left(Y_{i}\;is\;a\;structuralzero\right)$ is the structural zero rate and $\mu_{i}=E\left(Y_{i}|Y_{i}\;\mathrm{from the Poisson part}\right)$ is the mean of the Poisson part in the ZIP model. Unlike the ZIP model, the MZIP model aims to provide the overall mean of the outcome, i.e., $v_{i}=E\left(Y_{i}\right)=\left(1-\pi_{i}\right)\mu_{i},$ which can be modeled directly through


logvi=β0MZIP+β1MZIP·Intervention+βpMZIP∗AdditionalCovariates.

Therefore, the intervention effect on the overall mean is directly evaluated by $\beta_{1}^{MZIP}$ in the MZIP model.

We estimated two separate MZIP models for the outcomes at the 4-month and 15-month follow-ups. In each MZIP model, we evaluated history of high school drinking (0 = did not drink at all; 1 = drank alcohol), intervention group membership (0 = PFI; 1 = BMI), and their interaction term as predictors, while controlling for sex, race, first-year student status, and baseline number of drinks in a typical week. When the interaction between high school drinking and the intervention group membership did not significantly predict the outcome, we trimmed the interaction term to be parsimonious. The MZIP models were fit in R (R Core Team, 2022) using the *mcount* package (Zhou et al., 2022). Data used for the current study and the annotated R code are available on Mendeley data (http://doi.org/10.17632/vnpw693nnd.1; Tan et al., 2022).

## Results

**Figure 2:**
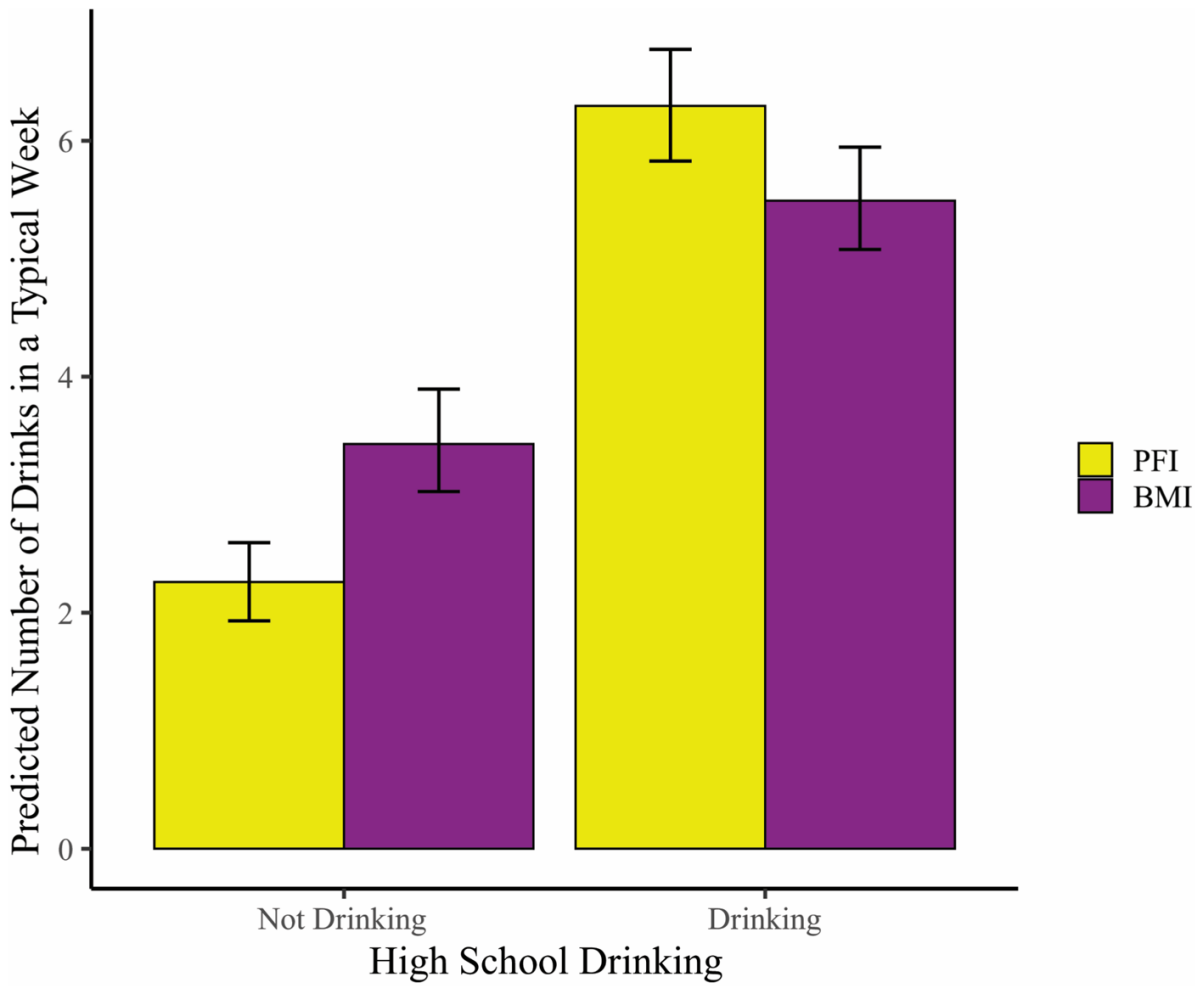
The interactions of high school drinking and intervention groups to predict the number of drinks in a typical week at the 4-month follow-up. The bar graph illustrates the significant interaction between high school drinking and intervention groups at the 4-month follow-up, as shown in Table 2. The error bar represents bootstrapped 95% confidence intervals. PFI, personalized feedback intervention; BMI, brief motivational intervention.

Descriptive statistics for the variables in the moderation analyses are shown in Table 1. Table 2 shows the results of the MZIP models at the 4-and 15-month follow-ups. Each MZIP model provides two sets of parameter estimates – one for predicting the overall mean number of drinks (left columns) and the other for predicting the excess zeroes part (right column; logit submodel) of the outcome. Being a male, White, and reporting a higher baseline number of drinks were significantly associated with a higher overall mean number of drinks at the 4-month follow-up. The interaction between high school drinking and intervention condition (BMI vs. PFI) was statistically significant in predicting the number of drinks in a typical week. The interaction effect can be interpreted as follows: based on the estimated parameter coefficients of high school drinking, intervention conditions, and their interaction, students without a history of high school drinking reported a 49% higher mean number of drinks when they received BMI (vs. PFI, with an estimated RR for BMI vs. PFI = 1.49 vs. 1). Conversely, for students with a history of high school drinking, the mean reported number of drinks in a typical week was 9% lower when they received BMI (vs. PFI, with an estimated RR for BMI vs. PFI = [1.49 × 0.61] vs. 1; hence 0.91 vs. 1). Figure 2, which shows the interaction effect, was derived based on the coefficients shown in Table 2. All individual values for predictors were retained in the equation while plugging in different values for high school drinking status (0 or 1) and intervention conditions (0 or 1). For the logit submodel (right column of Table 2), the predictors did not significantly predict non-drinking at the 4-month follow-up.

**Table 2:** Predicting the number of drinks in a typical week using MZIP models.

	Overall mean		Logit submodel (Predicting zero)	
	*RR*	95% CI	*OR*	95% CI
*4-month follow-up*				
Intercept	0.90	[0.63, 1.29]	1.50	[0.61, 3.72]
Male	1.51*	[1.29, 1.77]	0.62	[0.34, 1.14]
White	2.04*	[1.67, 2.50]	0.38*	[0.21, 0.67]
First-year student	1.13	[0.98, 1.31]	1.07	[0.58, 1.99]
Baseline number of drinks	1.03*	[1.02, 1.04]	0.96	[0.91, 1.01]
High school drinking	1.94*	[1.39, 2.71]	0.69	[0.28, 1.72]
BMI	1.49*	[1.01, 2.21]	0.56	[0.18, 1.75]
High school drinking × BMI	0.61*	[0.40, 0.93]	1.13	[0.29, 4.33]
*15-month follow-up*				
Intercept	2.38*	[1.91, 2.97]	1.31	[0.68, 2.52]
Male	1.60*	[1.41, 1.83]	0.61	[0.33, 1.12]
White	1.42*	[1.22, 1.65]	0.41*	[0.24, 0.73]
First-year student	1.37*	[1.23, 1.53]	1.26	[0.76, 2.09]
Baseline number of drinks	1.05*	[1.04, 1.05]	0.91*	[0.86, 0.96]
High school drinking	1.11	[0.91, 1.35]	0.39*	[0.21, 0.74]
BMI	0.77*	[0.69, 0.85]	1.37	[0.83, 2.27]

MZIP, Marginalized Zero-Inflated Poisson.

* *p* < 0.05.

At the 15-month follow-up, the interaction between high school drinking and intervention conditions was not statistically significant; the term was trimmed from the model. Table 2 shows that being a male, White, first-year student, and reporting a higher number of drinks at baseline were significantly related to a higher overall mean number of drinks at 15 months. BMI had a significant main effect on the overall mean number of drinks in a typical week. Specifically, those in the BMI condition had a 23% lower mean number of drinks at the 15-month follow-up than those in the PFI. For the logit submodel predicting no drinking, the odds of non-drinking were 61% lower among those who drank in high school, compared to those who did not.

## Discussion

The current study investigated whether high school drinking was associated with different intervention outcomes among students who received PFI compared to those who received BMI. Our findings suggest that alcohol consumption in high school may be a risk factor for continued drinking in college and demonstrate the potential to use high school drinking history to facilitate intervention allocation and development of more appropriate interventions for college students.

The results show that the effects of PFI and BMI on reducing alcohol use differed depending on students’ history of high school drinking. For students who did not drink in high school, those receiving PFI had fewer drinks at 4 months post intervention than those receiving BMI, which suggests that PFI might be a more effective short-term risk reduction strategy than BMI for lower-risk college students who just started drinking after entering college. Specifically, students who abstain from alcohol in high school may perceive greater salience of the negative consequences relating to the campus alcohol policy violation (Barnett et al., 2006; White et al., 2008), which serves to motivate them to change drinking behaviors (Morgan et al., 2008; Barnett et al., 2010). In this case, the in-person discussion of the feedback, a unique component of BMI designed to increase students’ motivation for change, may not be necessary; instead, PFI (as well as “getting caught”) can effectively reduce these students’ drinking. In addition, BMI has been found to have a larger effect among heavier drinkers than lighter drinkers (Murphy et al., 2001; Daeppen et al., 2011). For students without a history of drinking prior to college and who did not drink much when referred, personalized feedback that focuses on a few salient points is more likely to induce behavior change than when combined with in-person discussion (Ray et al., 2014). However, this explanation for the moderated effect will need to be further tested by future studies. Thus, college alcohol prevention/interventions would benefit from considering abstinence in high school to screen and select students to receive PFI rather than BMI for an immediate effect. Because PFI is inexpensive, quick, and easy to deliver, it allows college alcohol prevention efforts to reach a larger population faster at considerably lower costs.

We also found that students who drank during the senior year of high school consumed a greater number of drinks in college at the 4-month follow-up and were less likely to report no drinking at the 15-month follow-up across intervention groups, even after controlling for baseline drinking in college. These findings suggest that problematic drinking pathways may develop well before college and continue to evolve during college (Harford et al., 2002; White and Hingson, 2013). Structural and functional brain changes during adolescence may lead to increased reward-seeking risky behaviors, such as drinking (Bava and Tapert, 2010). Alcohol use, in turn, is associated with disruptions in normative adolescent brain development, such as irregular neural activities during executive functioning and decision-making (Silveri, 2012; Lees et al., 2020), which may further impair adolescents’ ability to restrain drinking. Because of adolescents’ heightened vulnerability to alcohol’s adverse effects, it would be important for preventative interventions to start early in high school and promptly identify students who are at higher risk for drinking. In addition to prevention/intervention strategies in high school, college prevention planning may use high school drinking history as one of the risk factors to screen and identify students to receive appropriate brief alcohol interventions early in college to reduce harm. Of note, previous research has provided initial evidence that students who were screened for risk in the senior year of high school and received a brief alcohol intervention in the freshman year of college reduced their drinking quantity and negative consequences of drinking (Marlatt et al., 1998; Baer et al., 2001).

The long-term effect of BMI at the 15-month follow-up was significantly stronger than PFI, suggesting an advantage of BMI over PFI that emerges over time (i.e., a sleeper effect; see White et al., 2007; Mun et al., 2009 for more details) possibly through increased motivation for change and self-efficacy over the long term (Magill et al., 2017). Because long-term intervention effects were better with BMI than with PFI, there may be an opportunity to track participants between 4 and 15 months to investigate why the initial greater gain from PFI over BMI for students who did not drink in high school was not found at 15 months. This will help continue research to identify strategies to maintain the advantageous outcome of PFI over BMI among students who do not drink before college for an extended period of time. The administration of booster sessions (Braitman and Henson, 2016; Braitman and Lau-Barraco, 2018) or an additional therapeutic component, such as a substance-free activity or relaxation training session (Murphy et al., 2019), may be beneficial. However, more work is required to understand the optimal timing, dosing, delivery methods, and audience to improve the benefits of PFI and brief alcohol interventions more broadly.

### Limitations and future directions

The current study provides evidence for the role that high school drinking plays in moderating the outcomes of two brief alcohol interventions (PFI vs. BMI) for college students. However, the findings should be interpreted in light of the following limitations. First, the data for the current study were collected from 2003 to 2006. The prevalence of past-year alcohol use among high school students has demonstrated a decline from the early-to mid-2000s (Miech et al., 2022). However, this decline in alcohol use prevalence should not affect the relations investigated in the current study because there is no theoretical foundation to suggest the prevalence of alcohol use would influence the outcomes of brief alcohol interventions. More recent studies also demonstrate that high school drinking is associated with college drinking (e.g., Scaglione et al., 2015; Lee et al., 2018b). Moreover, the two interventions studied, BMI and PFI, are rated as effective individual-level interventions and recommended by CollegeAIM (National Institute on Alcohol Abuse and Alcoholism, 2019) to be adopted on college campuses. Recent studies continue to investigate both interventions (e.g., Dunn et al., 2020; Gex et al., 2022; Li et al., 2022; Merrill et al., 2022; Tanner-Smith et al., 2022). Therefore, the current study’s findings have important clinical and practical implications that are of great interest to the alcohol research community.

Additionally, students retrospectively reported their alcohol use during their senior year of high school, so there might be recall errors. Furthermore, the senior year of high school is only 1 year, and may not be an accurate reflection of high school or “pre-college” drinking. Thus, future studies may consider the entire history of drinking, including intensity or manner of drinking, throughout the high school years. Next, the current study focused on high school drinking as a predictor of college intervention outcomes, but risky drinking behaviors may start even before high school. Early-onset of alcohol use and misuse at age 14 or younger is related to a higher likelihood of developing alcohol-related problems (Grant et al., 2001; Hingson et al., 2006). It would be important to investigate further whether a history of early onset alcohol use and misuse helps identify students for a more appropriate brief alcohol intervention during college.

Furthermore, the students in our sample were mandated students from a single university and primarily White, so the findings may not generalize across all young adults, including non-college students. In addition, students who were deemed at high risk were excluded from the study due to ethical and clinical considerations. Finally, we did not include a control group in the current study because all mandated students were required to receive an intervention based on university regulations. Future studies including an assessment-only control group may be helpful for examining the moderating effect of high school drinking on the efficacy of brief alcohol interventions.

In sum, the findings of the current study suggest high school drinking history could be used to screen and allocate students to a more appropriate alcohol intervention that meets their individual needs. A tiered intervention strategy may be feasible and promising if we further identify intervention effect modifiers or different responders to diverse brief alcohol intervention strategies. Given that both BMI and PFI are rated as effective individual-level interventions and endorsed by the National Institute on Alcohol Abuse and Alcoholism’s CollegeAIM to be implemented on college campuses (National Institute on Alcohol Abuse and Alcoholism, 2019), one promising tiered intervention strategy includes screening incoming college students for high school drinking history and selecting lower-risk students to receive PFI and higher-risk students to receive BMI.

## Data availability statement

The data and computing code used for this article are available at Mendeley Data: https://doi.org/10.17632/vnpw693nnd.1.

## Author contributions

LT, ZF, and E-YM contributed to the conception of the current study. HW was the PI on the study from which the data for this study were collected. LT, ZZ, DH, and E-YM contributed to the statistical analysis. LT and E-YM wrote the first draft of the manuscript. All authors contributed to manuscript revision and approved the submitted version.

## Funding

The project described was supported by grants R01 AA019511 and K02 AA028630 from the National Institute on Alcohol Abuse and Alcoholism (NIAAA). The data reported were collected with support from P20 DA017552 from the National Institute of Drug Abuse (NIDA). The content is solely the responsibility of the authors and does not necessarily represent the official views of the NIAAA, the NIDA, or the National Institutes of Health.

## Conflict of interest

The authors declare that the research was conducted in the absence of any commercial or financial relationships that could be construed as a potential conflict of interest.

## Publisher’s note

All claims expressed in this article are solely those of the authors and do not necessarily represent those of their affiliated organizations, or those of the publisher, the editors and the reviewers. Any product that may be evaluated in this article, or claim that may be made by its manufacturer, is not guaranteed or endorsed by the publisher.
