# Supplementary material for: Does abstaining from alcohol in high school moderate intervention effects for college students? Implications for tiered intervention strategies
Source: Front Psychol. 2022 Nov 30;13:993517. doi: 10.3389/fpsyg.2022.993517 (PMC9748095; doi:10.3389/fpsyg.2022.993517)
Supplement: Supplementary file 1 [file Table_1.pdf]

This supplemental material includes Table S1 and Appendix A. It is for Tan, L., Friedman, Z., Zhou, Z., Huh, D., White, H. R., & Mun, E.-Y. (2022). Does abstaining from alcohol in high school moderate intervention effects for college students? Implications for tiered intervention strategies. *Frontiers in Psychology*. https://doi.org/10.3389/fpsyg.2022.993517

Table S1.

Predicting the Number of Drinks in a Typical Week using MZIP models

	Overall mean		Logit submodel (Predicting Zero)	
	RR	95% *CI*	*OR*	95% *CI*
4-month follow-up				
Intercept	1.25	[0.97, 1.61]	1.44	[0.71, 2.93]
Male	1.59*	[1.35, 1.87]	0.51*	[0.29, 0.89]
White	1.89*	[1.56, 2.28]	0.55*	[0.31, 0.99]
First-year student	1.05	[0.90, 1.21]	1.90*	[1.04, 3.46]
Baseline number of drinks	1.02*	[1.02, 1.03]	1.00	[0.97, 1.05]
High school drinking[1]	1.12*	[1.06, 1.19]	0.71*	[0.58, 0.87]
BMI	1.05	[0.91, 1.22]	0.42*	[0.24, 0.74]
15-month follow-up				
Intercept	2.17*	[1.78, 2.65]	1.71	[0.86, 3.43]
Male	1.60*	[1.41, 1.81]	0.60	[0.34, 1.07]
White	1.30*	[1.12, 1.50]	0.59	[0.33, 1.05]
First-year student	1.35*	[1.21, 1.51]	1.18	[0.71, 1.96]
Baseline number of drinks	1.04*	[1.03, 1.05]	0.92*	[0.88, 0.97]
High school drinking[1]	1.10*	[1.05, 1.15]	0.64*	[0.49, 0.83]
BMI	0.81*	[0.73, 0.89]	1.08	[0.65, 1.79]

Notes. * $p < .05$. MZIP = Marginalized Zero-Inflated Poisson. [1] High school drinking = self-reported frequency of alcohol use during their senior year of high school (1 = did not drink at all to 8 = once a day or more). The interaction terms between high school drinking and the intervention group did not significantly predict the outcome at 4- and 15-month follow-up, and thus were trimmed.

Appendix A. Prototypical Personalized Feedback

ADAPS PERSONALIZED FEEDBACK

ID: nk2/25
Date: 4/30/13

Your Drinking In The 30 Days Prior To The Incident For Which You Were Referred To ADAPS

- According to the information you gave us, during the **HEAVIEST WEEK OF DRINKING** you drank:

  2 days per week

- During the **HEAVIEST WEEK OF DRINKING** you drank a total of:

  17 standard drinks

Standard Drink Equivalents

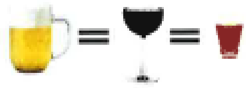

12 ounces   4 ounces   1 ounce

Your Recent Blood Alcohol Levels (BAL)

Below are estimates of your Blood Alcohol Levels (BAL) for your Peak Day of drinking in the 30 days prior to the incident (A), and your Peak BAL during your heaviest drinking week in the Past Month (B).

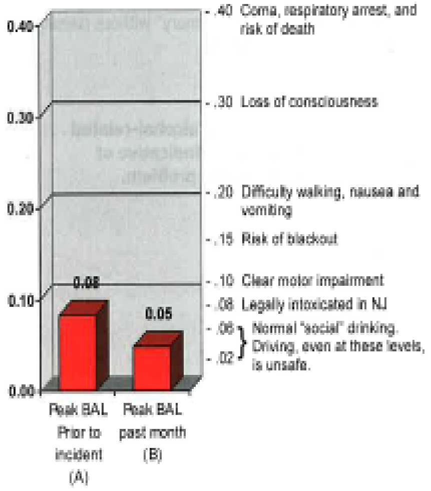

- **It would take approximately 6 hours for your Peak Blood Alcohol Level (BAL) in the 30 days prior to the incident to return to .00, and approximately 4 hours for your Peak BAL in the past month to return to .00.**

Heaviest and Typical Weekly Drinking

This graph shows you the number of drinks you drink per day in the heaviest week (blue) and a typical week (red) in the **past month.**

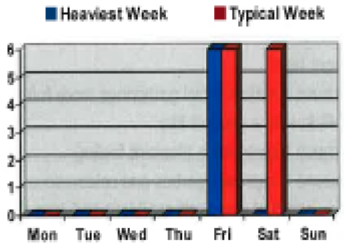

- **Compared to other college students, of your same gender, drinking 12 drinks in a typical week places you in the 80.5 percentile.**
- **You said 5% of students of your same sex drank more than you, but in reality you drink more than 80.5%.**
- **Most students think other students drink more than they actually do. Most Rutgers students drink 3 or fewer standard drinks when they drink.**

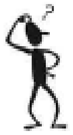

Did you know??

- A 12-ounce beer has between 132-168 calories.
- A "shot" (1.5 ounces) of 80 proof liquor has approximately 100 calories.
- A glass (5 ounces) of wine has approximately 100 calories.

In the past month, based on the number of drinks you consumed in a typical week, you are getting approximately the following amount of calories from alcohol:

6,000 calories

- **Over the month, it would require 1,014 minutes minutes of BRISK WALKING or 568 minutes of JOGGING to expend this number of calories.**
- **Based on your report of drinking 12 standard drinks, over a typical week, it would cost you approximately $180-$360 PER SEMESTER DEPENDING ON THE QUALITY OF BEVERAGES CONSUMED.**

Reasons For Drinking Alcohol

You indicated that **"most of the time"** or **"always"** you drank for the following reasons:

- To be sociable
- Because I like the feeling
- Because it's exciting
- To get high
- Because it makes social gatherings more fun
- To fit in with a group I like
- Because it gives me a pleasant feeling
- Because it improves parties and celebrations
- Because I feel more self-confident and sure of myself
- To celebrate a special occasion with friends
- Because it's fun

Does alcohol really do these things? Research suggests many of the social effects of alcohol are based on myths, placebo effects, and expectations we bring to the drinking situation.

Thoughts About Changing Your Alcohol Use

Within the **NEXT MONTH**, you indicated:

- **You are trying to change your drinking behavior.**

Alcohol-Related Problems

You reported that the following alcohol-related consequences had occurred at least once in the **LAST YEAR:**

- Not able to do your homework or study for a test.
- Acted bad or did mean things.
- Missed out on other things because you spent too much money on alcohol.
- Went to work or school drunk.
- Caused shame or embarrassment to someone.
- Neglected your responsibilities.
- Felt that you had a problem with alcohol.
- Missed a day (or part of a day) of school or work.
- Suddenly found yourself in a place that you could not remember getting to.
- Passed out or fainted suddenly.
- Had a fight, argument or bad feelings with a friend.
- Was told by a friend, neighbor or relative to stop or cut down on your drinking.
- Experienced nausea or vomiting.
- Had a hangover.
- Got into a fight.
- Had blackouts ("loss of memory" without passing out) as a result of drinking.

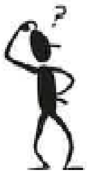

Did you know??

Having 5 or more alcohol-related problems may be indicative of having an alcohol problem.

Frequency of Drug Use

- You reported smoking 0 cigarettes PER DAY. Also, you estimated that 40% of college students smoked cigarettes in the past year. In the US, only 35.9% of male college students report having smoked cigarettes in the PAST YEAR.
- You reported using marijuana "0 days" in the PAST MONTH. Also, you estimated that 50% of college students used marijuana in the past year. In the US, only 37.3% of male college students report having used marijuana in the PAST YEAR.
- You reported using Cocaine or crack over the PAST YEAR. Also, in the PAST MONTH, you reported using this type of drug approximately 0 days.

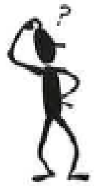

Did you know??

- **MDMA (Ecstasy):** In the US, only 5.3% of male college students report having used MDMA/Ecstasy/Ketamine/GHB in the **PAST YEAR.**
- **Amphetamines:** In the US, only 7.7% of male college students report having used amphetamines in the **PAST YEAR.**
- **LSD/HALLUCINOGENS:** In the US, only 10.5% of male college students report having used LSD/hallucinogens in the **PAST YEAR.**
- **COCAINE/CRACK:** In the US, only 6.2% of male college students report having used cocaine/crack in the **PAST YEAR.**
- **HEROIN/OPIATES:** In the US, only 0.4% of male college students report having used heroin/opiates in the **PAST YEAR.**

Drug-Related Problems

You reported that the following drug-related consequences had occurred at least once in the **LAST YEAR:**

- Not able to do your homework or study for a test.
- Acted bad or did mean things.
- Missed out on other things because you spent too much money on drugs.
- Went to work or school high.
- Caused shame or embarrassment to someone.
- Neglected your responsibilities.
- Friends, neighbors or relatives avoided you.
- Tried to control your drug use by trying to use only at certain times of the day or in certain places.
- Had withdrawal symptoms, (felt sick) because you stopped or cut down on drugs.
- Noticed a change in your personality.
- Drove shortly after taking drugs besides marijuana.
- Experienced nausea or vomiting after using.

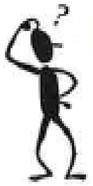

Did you know??

Having 3 or more drug-related problems may be indicative of having a drug problem.

Thoughts About Changing Your Drug Use

Within the **NEXT MONTH**, you indicated:

- **You are trying to change your drug use behavior.**

Risk Factors

There are certain behaviors, or pre-existing conditions that could put you at high risk for developing an alcohol or drug-related problem:

Behavior

- **You reported DRIVING AFTER DRINKING or USING DRUGS in the PAST YEAR.**

Alcohol-related accidents and violence are the leading cause of death among Americans under the age of 35. If you reported driving after drinking three or more drinks (or have ridden with a drunk driver), you have greatly increased your risk for injuries or legal problems.

- **You reported HAVING SEX WHEN YOU DIDN'T WANT TO while impaired by alcohol/drugs.**

If you're sexually active using a condom during sexual intercourse will protect you from HIV infection and sexually transmitted diseases (STD).

Family History

People with a history of alcohol or drug problems among their blood relatives have a higher risk for developing problems themselves. However, being aware of your drinking/drug use and making lower-risk decisions about alcohol or drug use now can lessen your risk of developing an alcohol or drug use problem in the future.

- **According to the information you provided, you HAVE A POSITIVE FAMILY RISK FACTOR for alcohol or drug use problems.**

Depression

Depression is a medical condition that can often lead to increased drinking or drug use in order to deal with the symptoms. Many people who are experiencing problems with alcohol and drug may also feel depressed as a result of their substance use. It is important to remember that treatment is available for those suffering from depression. A score of 11 or above on the Beck Depression Inventory is considered significantly elevated and one may consider talking to a professional to discuss options for the treatment of depression.

- **Your Beck Depression Score was: 0**

Protective Factors

These are some things you reported doing which help in avoiding negative consequences from drinking:

- Chose not to drink alcohol.
- Used a designated driver.
- Ate before and/or during drinking.
- Kept track of how many drinks I was having.
- Paced my drinks to 1 or fewer per hour.
- Avoided drinking games.

Here are some other strategies that you might want to try to reduce the negative effects of drinking:

- Switch between alcoholic and non-alcoholic beverages.
- Determine, in advance, not to exceed a set numbe of drinks.
- Have a friend let you know when you have had enough.
- Drink an alcohol look-alike (non-alcoholic beer, punch).
